# Clinical Performance and Patients’ Satisfaction of Digitally Versus Conventionally Fabricated Dentures: A Randomized Controlled Pilot Study

**DOI:** 10.3390/dj14040221

**Published:** 2026-04-09

**Authors:** Jonas Rechlin, Florian Beuer, Robert Nicic, Rebecca Noetzel, Wolfgang Hannak, Elisabeth Prause

**Affiliations:** Department of Prosthodontics, Geriatric Dentistry and Craniomandibular Disorders, Charité-Universitätsmedizin Berlin, Aßmannshauser Str. 4-6, 14197 Berlin, Germany; jonas.rechlin@charite.de (J.R.); florian.beuer@charite.de (F.B.); robert.nicic@charite.de (R.N.); rebecca.noetzel@charite.de (R.N.); wolfgang.hannak@charite.de (W.H.)

**Keywords:** dentures, CAD/CAM, milling, oral health-related quality of life, digital

## Abstract

**Background/Objectives:** Although digital workflows for complete denture fabrication are increasingly implemented in clinical practice, randomized controlled pilot trials directly comparing their clinical performance and patient satisfaction with conventional complete dentures (CCDs) remain scarce. This study aimed to compare patient satisfaction and clinical effectiveness between conventionally and digitally fabricated complete dentures (DCDs). **Methods:** In the present exploratory randomized controlled clinical pilot study using a cross-over design, 15 edentulous patients received both a conventionally fabricated and a digitally fabricated complete denture in randomized order. Each denture was worn for a three-month adaptation period. Patients were blinded to the fabrication method. Oral health-related quality of life was assessed using the OHIP-G49 questionnaire, and clinical performance was evaluated using standardized criteria at baseline, after three months with the first denture, and after three months with the second denture. **Results:** Both fabrication methods yielded satisfactory clinical outcomes in all patients. All 15 patients rated the DCDs as highly satisfactory, while 14 patients rated the CCDs equally favorably; one patient was unable to tolerate the conventional denture. DCDs demonstrated a slight but consistent advantage in oral health-related quality of life (OHRQoL) scores. **Conclusions:** Both conventional and digital complete dentures are clinically effective and well accepted by edentulous patients. However, DCDs offer a modest improvement in patient satisfaction and OHRQoL. Digitally fabricated complete dentures provide comparable clinical results to conventional methods while offering potential advantages in patient comfort and perceived quality of life. Given the exploratory nature of the study and the limited sample size, the results should be interpreted with caution and primarily serve to inform future, adequately powered randomized clinical trials.

## 1. Introduction

Currently, complete dentures can be fabricated using either conventional or digital methods. In recent years, computer-aided design (CAD) and computer-aided manufacturing (CAM) have become integral processes in dentistry [1], contributing substantially to the development of novel clinical workflows [2]. Digital workflows have been increasingly integrated into clinical dental practice, particularly in prosthodontics. Compared to conventional manufacturing techniques, CAD/CAM systems offer numerous advantages, including increased productivity, reduced costs, more efficient data management, and shorter treatment times. These developments benefit both dental professionals and patients [3,4,5,6].

The digital fabrication of complete dentures has been investigated since the 1980s [3,7,8]. Today, the production of such dentures is typically performed using subtractive manufacturing methods. However, current evidence regarding patient satisfaction with digitally fabricated complete dentures remains inconsistent. While some studies report higher levels of satisfaction among patients receiving CAD/CAM dentures compared to those with conventionally fabricated ones [9,10,11,12], other investigations suggest that overall patient satisfaction is comparable between the two groups [3,13,14,15,16]. The current scientific evidence regarding the clinical performance of digitally fabricated dentures is heterogeneous and limited, with a notable absence of direct comparisons to conventionally manufactured complete dentures.

The impact on the patient’s quality of life and patient acceptance and compliance must be taken into consideration when evaluating the overall quality of a prosthetic dental rehabilitation. The OHIP-G49 constitutes a standardized assessment instrument developed by Slade and Spencer [13,17]. Through structured interviews conducted with 64 Australian dental recall patients, the authors derived 49 items categorized into seven distinct subscales for the comprehensive evaluation of patients’ oral health conditions and associated difficulties [13,17]. Elevated OHIP-G49 summary scores, at both the total scale and subscale levels, indicate more severe oral health impairments. The psychometric properties of the OHIP-G49, including validity and reliability, have been extensively validated through epidemiological and cross-cultural investigations [13,18,19,20,21,22].

The clinical performance of DCDs has been scientifically evaluated. Laboratory studies indicate that CAD-CAM denture bases demonstrate improved fit [23,24] and retention relative to conventional prostheses [24,25], potentially minimizing traumatic ulcer formation [23,24]. Evidence regarding reduced monomer release from prepolymerized resins [24,26] and enhanced fracture resistance of CAD-CAM bases [24,27] remains insufficient. Clinical studies evaluating CAD-CAM dentures are predominantly limited to case reports and case series [24,28,29,30,31,32,33,34], demonstrating technical feasibility and workflow protocols. The most frequently reported clinical issues involved esthetic adjustments and pressure relief [24]. One randomized controlled trial (*n* = 15) compared DCDs and CCDs fabricated by supervised dental students [24,35]. Faculty assessments favored DCDs for base contour, extension, stability, retention, and overall quality [24,35]. After one week of wear, participants significantly preferred digital prostheses for retention, function, comfort, technical efficiency, and acceptability. Neither group was blinded to the fabrication method [24,35].

Despite increasing clinical use of CAD/CAM dentures, high-quality randomized controlled cross-over pilot trials evaluating both patient-reported outcomes and standardized clinical performance within the same individuals remain limited. Furthermore, evidence specifically addressing milled digital dentures fabricated under controlled clinical conditions is scarce. Therefore, the present pilot study aimed to compare OHRQoL and clinical performance of conventionally fabricated and digitally milled complete dentures using a randomized cross-over design.

The null hypothesis of the present study was that there would be no significant difference between digitally and conventionally fabricated complete dentures with respect to OHRQoL and clinical performance.

## 2. Materials and Methods

### 2.1. Study Design and Population

This study was approved by the Ethics Committee under application number EA1/183/16. Furthermore, the study was retrospectively registered in the German Clinical Trials Register (DRKS00039355) on 18 February 2026. At the time of study initiation (2015), prospective registration of investigator-initiated pilot trials was not yet consistently implemented in our institution. The primary aim of this exploratory pilot study was to generate preliminary clinical data and assess feasibility rather than to confirm predefined hypotheses. Importantly, the study protocol, inclusion criteria, outcome measures (OHIP-G49 and clinical evaluation), and analysis plan were defined prior to patient recruitment and remained unchanged throughout the study period. No modifications to study design, endpoints, or statistical analysis were made after data collection. The retrospective registration was performed to ensure transparency and compliance with current reporting standards.

The study included 15 participants aged 58–83 years (mean 69.8 ± 8.7; median 69). The treatment period extended from 2015 to 2025, with prosthetic rehabilitation of the final five patients scheduled for completion in the first quarter of 2025. The extended recruitment period was attributable to the cross-over study design, which required fabrication and evaluation of two complete dentures per patient, defined three-month adaptation periods for each prosthesis, and the limited availability of eligible edentulous patients willing to undergo both treatment modalities during the coronavirus pandemic when no clinical treatment was allowed.

Participants were recruited from the Department of Prosthodontics at Charité–Universitätsmedizin Berlin. All 15 individuals received verbal and written information about the study and provided written informed consent prior to enrollment.

This study was designed as a randomized controlled cross-over clinical pilot trial. A cross-over design was chosen to enable within-subject comparison of both denture fabrication methods, thereby minimizing interindividual variability in oral health-related quality of life and clinical performance assessments. No formal a priori sample size or power calculation was performed, as the primary aim was to generate preliminary clinical data regarding patient-reported and clinical outcomes of digitally and conventionally fabricated complete dentures. The sample size was determined pragmatically based on patient availability, the extensive clinical protocol, and the long adaptation period required for each participant.

Participants were randomly allocated to one of two treatment sequences using a computer-generated randomization list: (1) conventionally fabricated complete dentures (CCD) followed by digitally fabricated complete dentures (DCD), or (2) DCD followed by CCD. Each denture was worn for a period of three months to allow functional adaptation before cross-over to the alternative prosthesis. The second complete denture was fabricated after completion of the three-month wearing period of the first denture. Both dentures were therefore not fabricated simultaneously. This sequential fabrication ensured that each prosthesis was manufactured and adjusted independently under comparable clinical conditions.

Participants differed in their previous prosthetic status, ranging from complete dentures in both jaws and single-arch complete dentures to no prior prosthetic rehabilitation. The sample comprised 7 men (46.7%) and 8 women (53.3%) (Table 1). All patients were informed about the study design, and written informed consent was obtained. Following agreement to participate in the study, a thorough dental examination was performed. The degree of alveolar ridge resorption ranged from moderate to severe and was classified according to the Cawood and Howell classification (Classes III–V), a widely accepted system for describing residual ridge morphology in edentulous patients [36].

Only patients with a need for complete dentures in the upper and lower jaw were included in the present study. Inclusion criteria comprised willingness to participate and wear both denture designs, adequate physical and cognitive ability to evaluate the prostheses, and written informed consent. Exclusion criteria included known allergies to prosthetic materials, severe xerostomia, pronounced muscular dysfunction, or systemic conditions likely to interfere with study participation.

The present randomized controlled cross-over clinical pilot trial was reported in accordance with the Consolidated Standards of Reporting Trials (CONSORT) statement and its extension for randomized cross-over trials (Table S1) [37].

Patients were blinded to the type of denture fabrication throughout the study period. Questionnaire data were anonymized prior to statistical analysis, and the statistician had no access to identifiers related to denture type. Clinicians involved in prosthesis fabrication and clinical evaluation were not blinded.

The random allocation sequence was generated by an independent study nurse. Sequence generation was performed using a computer-based random number generator (R statistical software, version 4.0.3, R Foundation for Statistical Computing, Vienna, Austria), applying simple randomization with a 1:1 allocation ratio to one of two treatment sequences (CCD→DCD or DCD→CCD). No stratification or blocking procedures were implemented due to the exploratory pilot character and limited sample size.

Personnel responsible for patient enrollment had no access to the randomization list. The allocation sequence remained concealed from enrolling clinicians until assignment. Denture fabrication and treatment procedures were conducted according to the assigned sequence. Patients were blinded to the denture fabrication method, whereas treating clinicians could not be blinded due to the inherent differences between workflows.

### 2.2. Intervention

**Figure 1:**
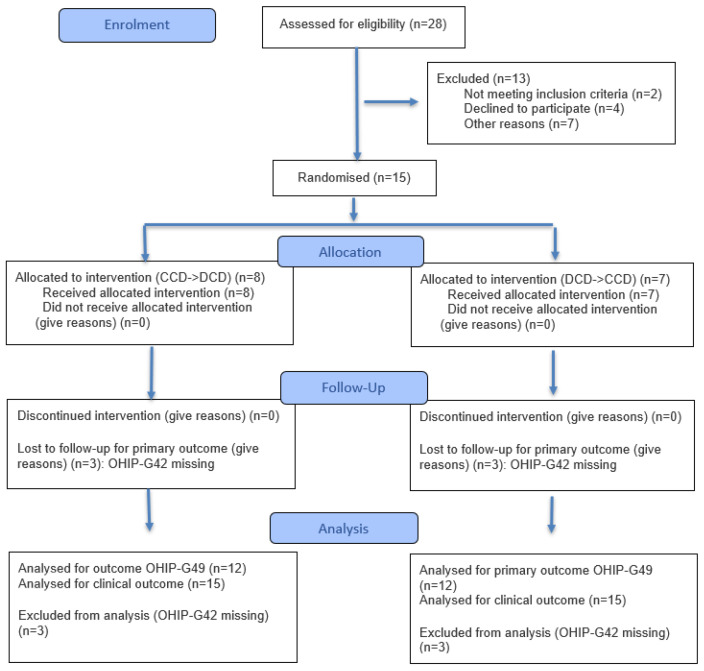
CONSORT 2025 Flow Diagram of the present clinical pilot study.

Each patient received two complete dentures in randomized order: one fabricated using conventional analog techniques and one produced digitally following the Digital Denture protocol (Ivoclar, Schaan, Liechtenstein). The study was divided into two phases. Initially, patients were provided with the first complete denture (either analog or digital). Following a three-month wearing period, the second denture was fabricated and delivered (Figure 1). Both dentures (analog and digital) were manufactured by a single highly experienced dental technician (Figure 1).

**Figure 2:**
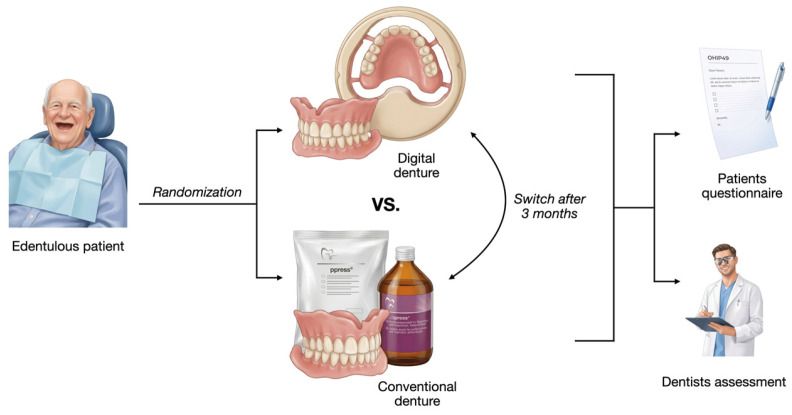
Study design of the randomized crossover clinical pilot trial. Edentulous patients were randomly allocated to receive either a digitally fabricated complete denture or a conventionally fabricated complete denture. After a three-month wearing period, patients switched to the alternative denture. Patient-reported outcomes were assessed using questionnaires, and clinical performance was evaluated by dentists.

Throughout the recruitment and treatment period, the digital denture workflow followed a consistent milling-based fabrication concept. The same fundamental clinical protocol, materials, and manufacturing principles were applied for all digitally fabricated dentures. While routine updates of hardware and software occurred over time as part of standard laboratory maintenance, no fundamental changes to the digital workflow, materials, or fabrication concept were implemented that would compromise the comparability of the digitally fabricated dentures (Figure 2).

The fabrication of CCDs was completed over six appointments. The preliminary impressions, functional impressions, preliminary jaw relation record, and vertical and horizontal definitive jaw relation were conducted separately. Subsequently, the wax try-in of the dentures and final delivery were performed.

In contrast, the fabrication of DCDs required four appointments: preliminary impressions, preliminary jaw relation and facebow; functional impressions and definitive jaw relation; recording of mandibular movements; try-in dentures; and delivery of definitive dentures.

In both groups, evaluation of the prostheses was conducted by the study supervisor during the follow-up visit. OHRQoL was assessed using the OHIP-G49 questionnaire.

### 2.3. Fabrication of Conventional Complete Dentures (CCDs)

**Figure 3:**
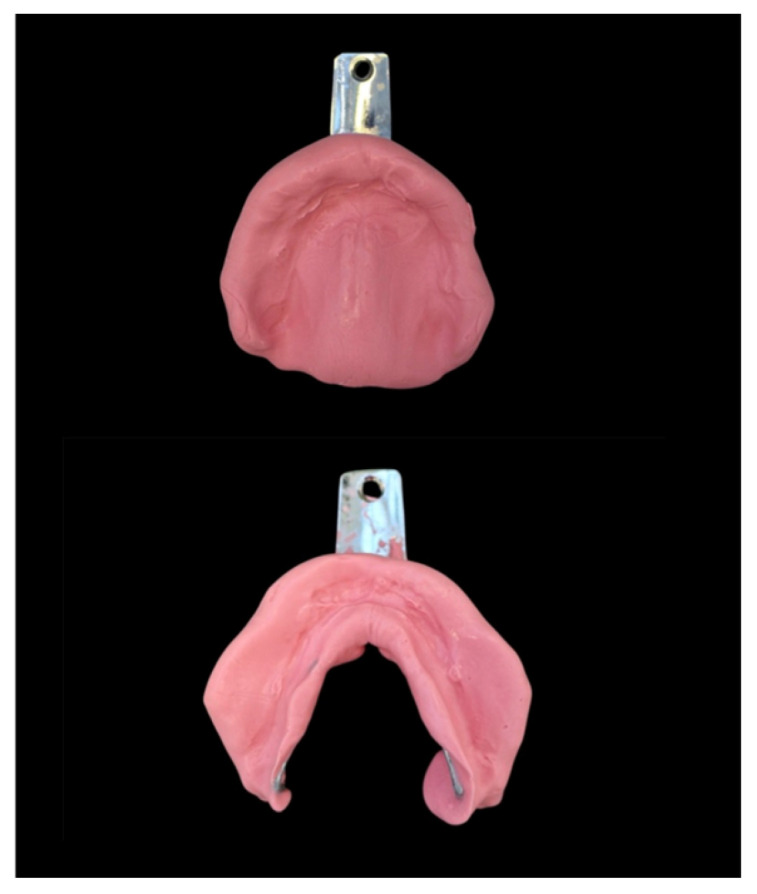
Preliminary impressions with Schreinemakers trays and alginate.

Preliminary impressions were taken using Schreinemakers impression trays and alginate (Omnident, Rodgau, Germany) (Figure 3).

**Figure 4:**
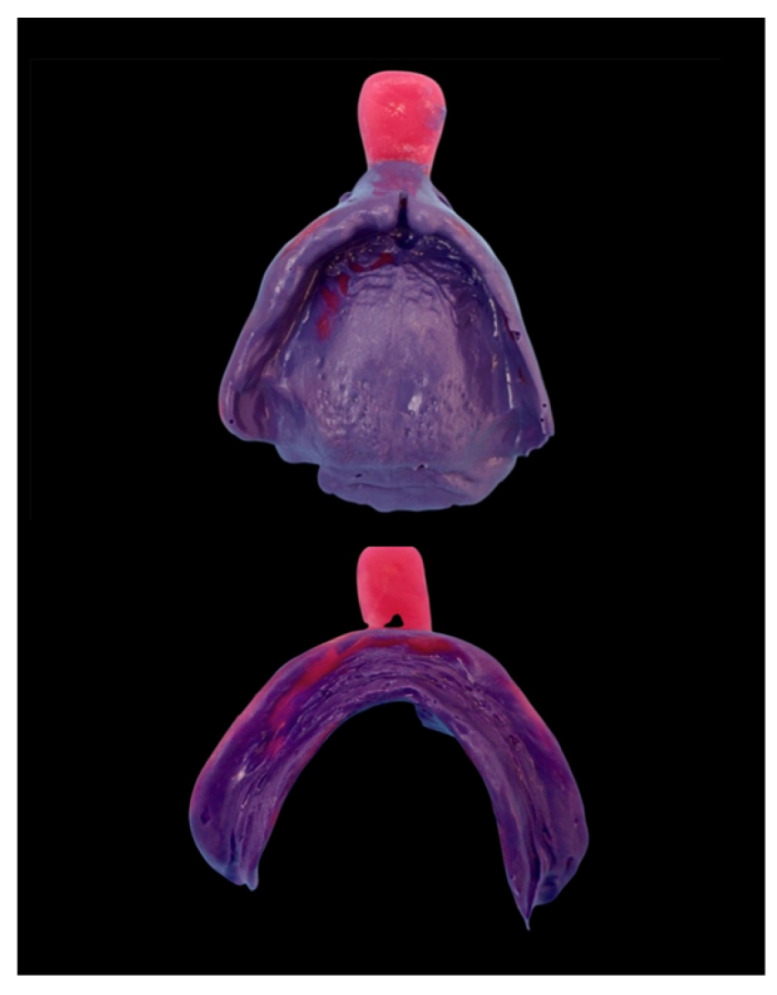
Functional impression.

Preliminary bite registration was conducted (Centric Tray, Ivoclar). The dental technician fabricated preliminary models using dental stone and mounted them provisionally based on the preliminary bite registration. Custom trays made from light-curing resin (Light Curing Base Plates, Henry Schein, New York, NY, USA) were used to fabricate individual impression trays for conducting the functional impression using polyether impression material (Impregum, 3M, Seefeld, Germany) (Figure 4).

**Figure 5:**
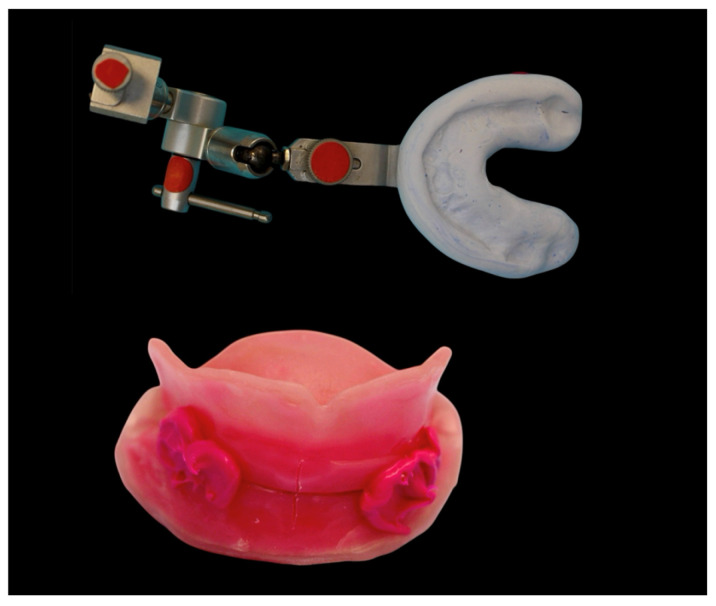
Definitive jaw relation record and face bow transfer.

The definitive jaw relation record was obtained using conventional methods with stabilized record bases and wax rims. The vertical dimension of occlusion was determined clinically using wax ridges that were aligned according to the Camper’s plane and the bipupillary line plane. Furthermore, jaw relation was based on facial proportions and interocclusal rest space. Subsequently, an arbitrary facebow transfer (Artex, Amann Girrbach, Pforzheim, Germany) was performed to relate the maxilla to the articulator hinge axis. The bite fork was positioned on the maxillary record base intraorally and stabilized, while the facebow frame was aligned according to anatomical reference points. The bite fork was then transferred to the articulator to mount the maxillary cast in an anatomically oriented position. The final interocclusal record was made using silicone registration material (Futar D, Kettenbach Dental, Eschenbach, Germany) (Figure 5).

**Figure 6:**
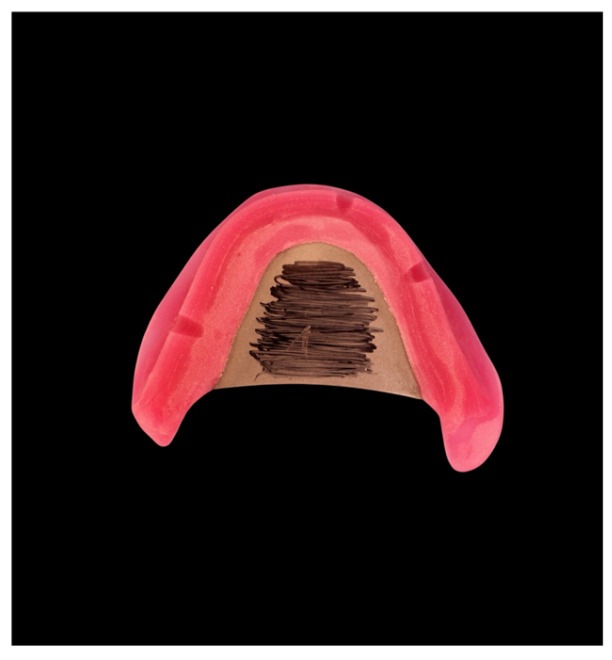
Horizontal jaw relation.

The dental technician fabricated esthetic record bases from light-curing resin, incorporating wax rims for jaw relation recording. The fit of these bases in the patient’s mouth was verified. Patients were asked to perform standardized mandibular movements, including repeated protrusive and lateral excursions (left/right) guided by the clinician to support centric relation registration (Figure 6). The patient and clinician jointly selected the tooth shape and shade.

**Figure 7:**
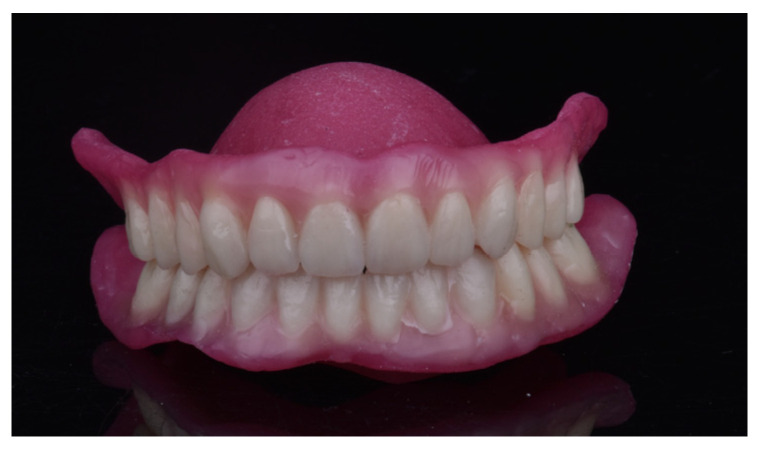
Wax try-in.

Subsequently, a wax try-in was performed to assess esthetics and jaw relation. Phonetic verification was performed by assessing /s/ and /z/ sounds (counting from 60 to 70) to evaluate the closest speaking space and /f/ and /v/ sounds to verify incisal edge position relative to the lower lip. Speech clarity and patient comfort were assessed qualitatively, and adjustments were performed if needed (Figure 7).

**Figure 8:**
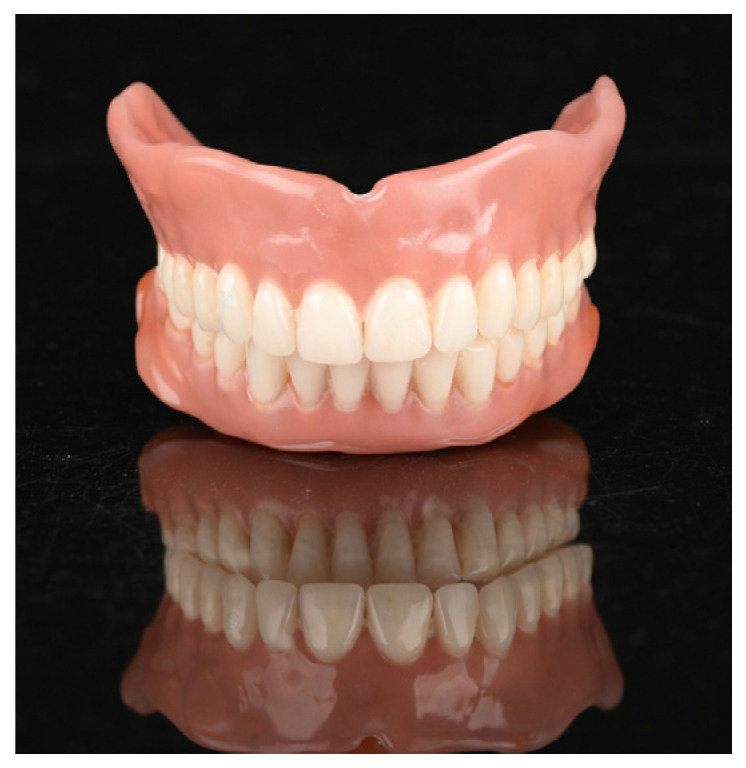
Delivery of definitive CCDs after processing.

Upon successful completion of this treatment step, dentures were processed in acrylic. For the fabrication of CCDs, the technician used polymethylmethacrylate (PMMA) by mixing powder with monomer (PalaXpress, Kulzer, Hanau, Germany). Polymerization was carried out in a pressure pot. A follow-up examination was conducted after one week of clinical function (Figure 8).

Both interventions (CCD and DCD) were delivered according to predefined standardized clinical and laboratory protocols. All dentures were fabricated by a single experienced dental technician to ensure procedural consistency. Clinical treatment steps, adjustments, and follow-up evaluations were performed under supervision within the same clinical setting. Adherence to the allocated intervention sequence was complete for all participants, except for one patient who was unable to tolerate the conventional denture. Overall, interventions were delivered as intended without relevant protocol deviations.

No relevant differences in concomitant care occurred between intervention phases. All patients received identical oral and denture hygiene instructions, as well as comparable recall and adjustment appointments during the adaptation periods. No additional prosthetic or adjunctive treatments influencing study outcomes were provided.

### 2.4. Fabrication of Digital Complete Dentures (DCD)

**Figure 9:**
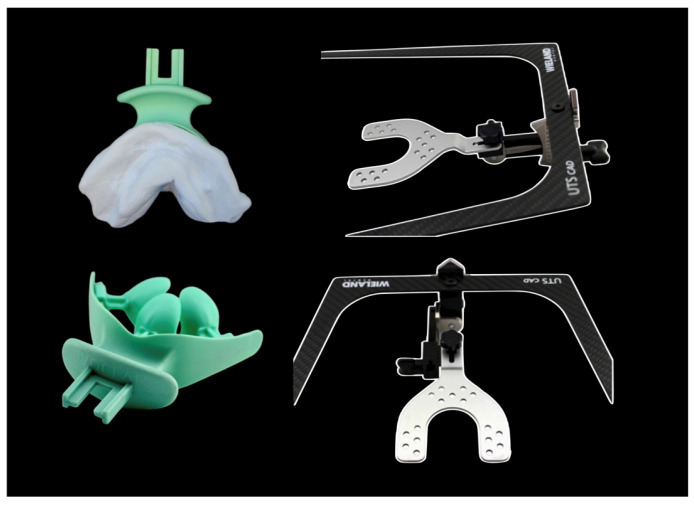
Preliminary jaw relation and digital jaw relation using UTS CAD (Ivoclar).

The manufacturing of DCDs was conducted as follows. Impressions were obtained conventionally as described above for CCDs. Scanning of these impressions, along with the preliminary bite registration, was performed using a laboratory scanner (D2000, 3Shape, Copenhagen, Denmark). The analog provisional jaw relation (Centric Tray, Ivoclar) was incorporated using impression holders (Ivoclar). The jaw relation was recorded digitally (UTS CAD, Ivoclar). Furthermore, the angles of the occlusal plane in relation to Camper’s plane and the bipupillary line were recorded digitally (UTS CAD, Ivoclar). These measurements were transferred into the design software module (Digital Denture Professional, Version 19, 3Shape, Copenhagen, Denmark) to establish the virtual orientation of the occlusal plane for denture design. The centric condylar position was determined individually via intraoral gothic arch tracing (Gnathometer CAD) (Figure 9).

**Figure 10:**
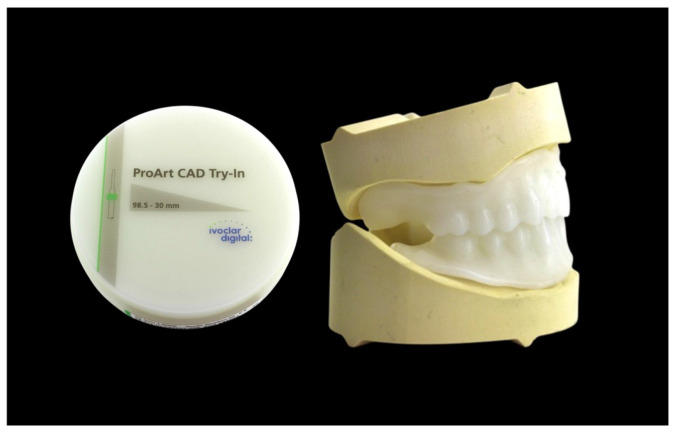
Three-dimensional bite plates before functional impression.

**Figure 11:**
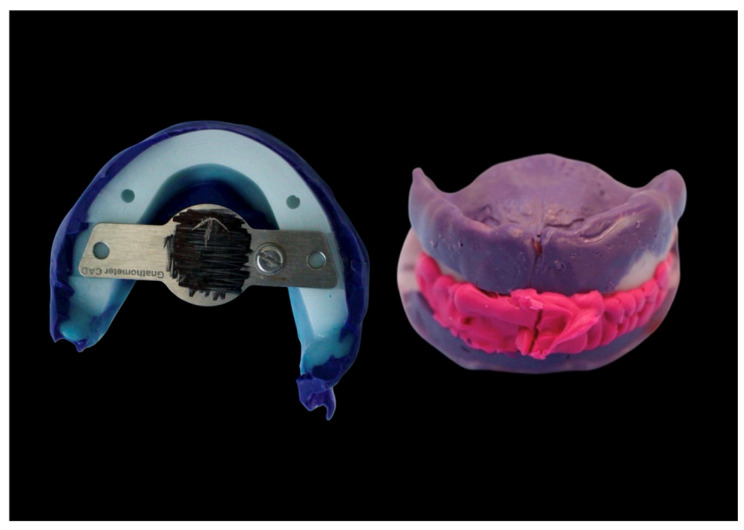
Horizontal jaw relation using Gnathometer CAD and functional impression.

Try-in dentures and 3D bite plates were milled from opaque white PMMA disks (ProArt CAD Try-In, Ivoclar) and combined with the functional impression (Figure 10 and Figure 11).

**Figure 12:**
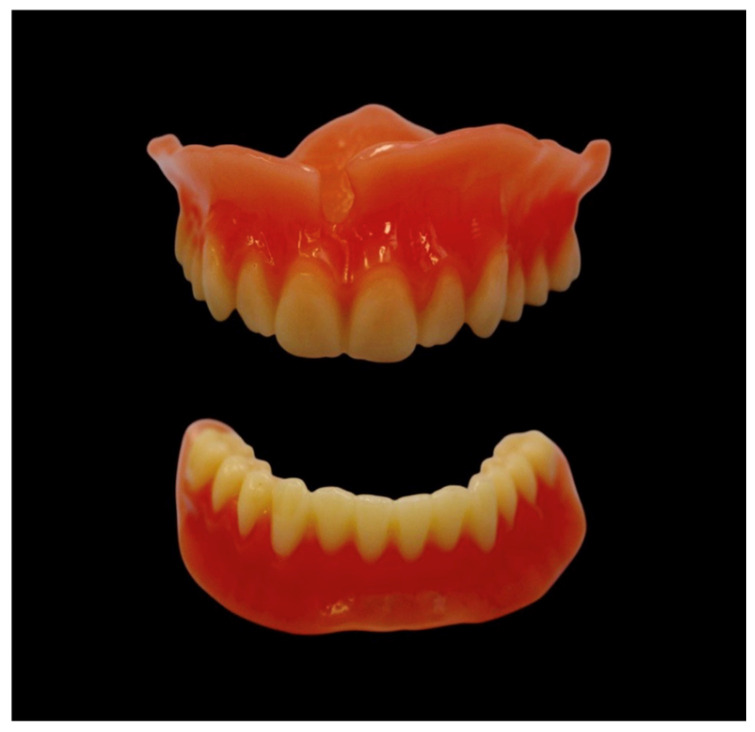
Final complete dentures before insertion.

Denture teeth were milled from tooth-colored double-crosslinked disks (SR Vivodent CAD, Ivoclar). Patients could choose from the following tooth shades: BL3 (Bleach), A1, A2, A3, A3.5, B1, B3, C2, and D2 (VITA Zahnfabrik, Bad Säckingen, Germany). The denture bases were fabricated using gingiva-colored PMMA disks (Ivobase CAD, Ivoclar), with four base shades available for selection. Bonding of the denture base to the tooth arch was achieved using a self-curing two-component adhesive (Ivobase CAD Bond, Ivoclar). Milling procedures were conducted using a milling machine (PrograMill PM7, Ivoclar) (Figure 12).

CCDs and DCDs were delivered to the patients with oral and denture hygiene instructions. Recall sessions were conducted individually to adjust the dentures during the initial days and weeks.

### 2.5. Oral Health Impact Profile (OHIP-G49)

Patients’ subjective assessments of the dentures were conducted using the German version of the Oral Health Impact Profile (OHIP-G49), with item allocation based on the subscales defined in the original English version. The OHIP-G49 questionnaire was administered under standardized and supervised conditions. All patients received identical instructions prior to completion. If clarification of individual items was requested, neutral explanations were provided without influencing the patients’ responses. Questionnaires were completed in a calm clinical setting to minimize distraction and incomplete responses. Completion of the OHIP-G49 questionnaire required approximately 10–15 min per patient. Some participants requested clarification of individual items, which was provided in a standardized manner to ensure consistent understanding.

The questionnaire covered the following dimensions: functional limitation, pain, psychological discomfort, physical disability, psychological disability, social disability, and handicap. Evaluation was based on a 5-point Likert scale, where higher scores indicated a greater negative impact on oral health-related quality of life. The total possible score ranged from 0 to 245. Patients completed the OHIP-G49 questionnaire at three time points: prior to treatment, after three months of wearing the first set of dentures, and after an additional three-month period wearing the second set. At no point were patients informed about which type of denture they were using. The order of denture insertion was randomized to ensure unbiased responses (Table 2).

### 2.6. Clinical Evaluation of Dentures (CCD and DCD)

The complete dentures were evaluated by the study supervisor using 13 established clinical criteria: retention, static and dynamic occlusion, muscle control, base contour, tooth positioning, fit, esthetics, lip support, vertical dimension, extension, stability, and phonetics (Table 3) [38,39]. Each criterion was rated on a 5-point scale, with higher scores indicating poorer performance (total score: 0–52). The scoring system for the evaluation of complete dentures according to objective dental criteria was as follows: 0 = very good, no negative findings; 4 = remake required, clinically unacceptable. Assessments were conducted at the follow-up appointment 1–2 weeks after insertion. All clinical assessments were performed by a single experienced examiner. Although this approach reduces inter-examiner variability, examiner blinding was not feasible due to obvious differences between workflows and materials. Therefore, observer bias cannot be fully excluded.

The total evaluation score was categorized into groups (Table 4). Data were collected three months after the insertion of each complete denture.

Post-insertion adjustments were conducted in both CCD and DCD groups during the initial follow-up period. A three-month adaptation period was implemented between both interventions to minimize potential carry-over effects. Due to the long accommodation period, clinically relevant carry-over effects were assumed to be negligible. Nevertheless, outcomes were always collected at the end of each intervention phase after completion of the standardized adaptation period.

**Table 4:** Categorization of overall denture evaluation scores into three quality levels (highly satisfactory, satisfactory with limitations, unsatisfactory), based on the sum of objective dental criteria.

Score	Evaluation Category
0–17	Highly satisfactory
18–34	Satisfactory with limitations
35–52	Unsatisfactory

### 2.7. Statistical Analysis

Statistical analyses were performed using R (version 4.5.1). Prior to inferential analysis, the distribution of continuous variables was assessed using the Shapiro–Wilk test and visual inspection of Q–Q plots. Due to the small sample size and the absence of normal distribution for several variables, non-parametric statistical methods were applied. Continuous variables are therefore reported as median and interquartile range (IQR), while categorical variables are presented as absolute frequencies and percentages. Changes in OHIP-G49 scores over time were analyzed using Friedman tests for related samples, followed by pairwise Wilcoxon signed-rank tests with Holm correction. Comparisons between CCDs and DCDs were conducted using Wilcoxon signed-rank tests. Effect sizes were calculated as Kendall’s W for Friedman tests and as r for Wilcoxon tests. α = 0.05. As no significant differences were observed between CCDs and DCDs, no claims of superiority were made.

## 3. Results

Of the 15 included patients (8 female, 7 male), OHIP-G49 values at time point t2 were unavailable for 2 patients (13.3%) due to non-response. Therefore, 12 patients with complete datasets across all three time points and 13 patients with complete OHIP-49 datasets were analyzed for the inferential statistical analyses of OHIP-49 trajectories. For dental evaluations, complete datasets were available for all 15 patients. Baseline demographic and clinical characteristics are presented in Table 1. No relevant differences between randomized treatment sequences were observed.

Analyses were based on data availability at each time point. Complete OHIP-G49 datasets were available for 12 participants longitudinally, while dental evaluation data were complete for all 15 participants. Outcomes are reported as medians and interquartile ranges. Statistical analyses included Friedman tests, Wilcoxon signed-rank tests, and corresponding effect sizes. No statistically significant differences between CCDs and DCDs were detected.

**Figure 13:**
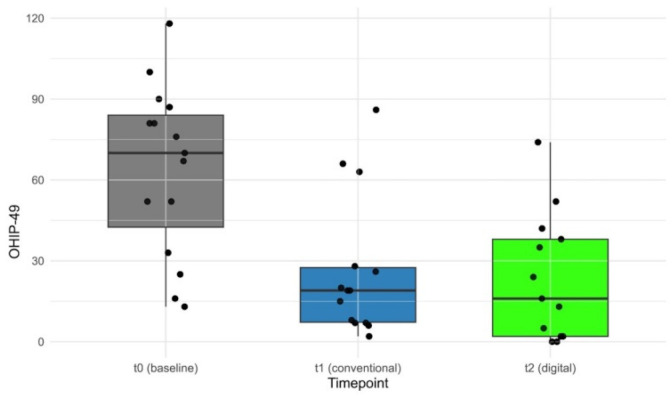
A boxplot representation of OHIP-G49 values at the three measurement time points t0 (pre-treatment), t1 (conventional prosthesis), and t2 (digital prosthesis). Individual data points are overlaid as dots. Boxplots illustrate median values and interquartile ranges.

**Figure 14:**
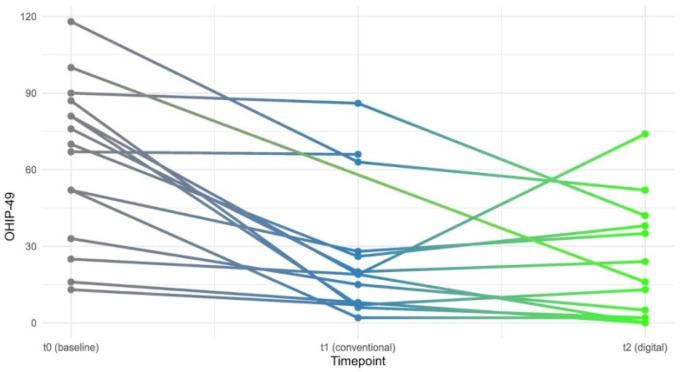
Line graph of individual patient trajectories across the three measurement time points. Each line represents one patient. The color gradient from gray (t0) through blue (t1) to neon green (t2) visualizes temporal progression. Missing values result in interrupted lines.

The OHIP-G49 values, where lower values correspond to better oral health-related quality of life, showed clear changes across measurement time points. At t0 (pre-treatment), the median was 70.0 with an IQR of 41.5, ranging from 26.0 to 126.0 (*n* = 15). At t1 (conventional prosthesis), the median decreased to 19.0 with an IQR of 20.2, ranging from 0.0 to 78.0 (*n* = 14). At t2 (digital prosthesis), the median was 16.0 with an IQR of 36.0, ranging from 0.0 to 70.0 (*n* = 13). The median OHIP-G49 value decreased from t0 to t1 by 51.0 points and from t1 to t2 by an additional 3.0 points (Figure 13 and Figure 14).

**Figure 15:**
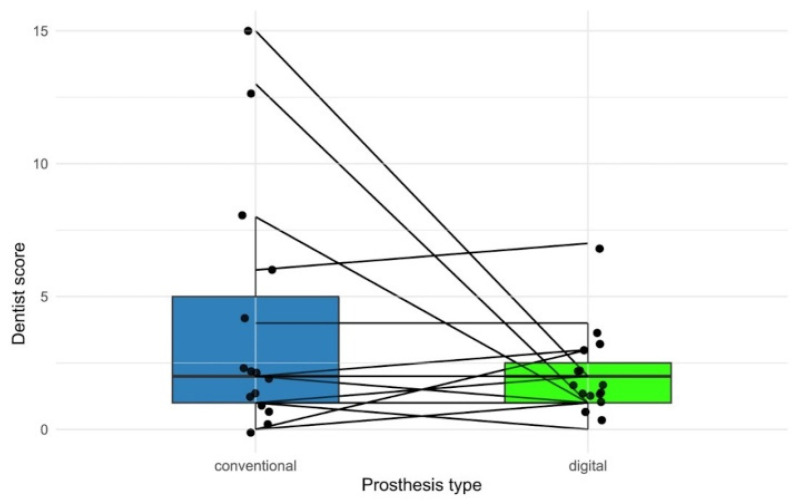
Paired representation of dental evaluation for conventional and digital prostheses. Boxplots show the distribution, while overlaid points show individual values. Connecting lines between groups represent pairwise comparisons for each patient.

The dental evaluation, where lower values correspond to better clinical assessment, showed that CCDs had a median of 2.0 with an IQR of 4.0, ranging from 0.0 to 15.0 (n = 15), while DCDs had a median of 2.0 with an IQR of 1.5, ranging from 0.0 to 6.0 (n = 15). The median difference between evaluations was 0.0 points, with DCDs showing less variability in evaluations (Figure 15).

The Friedman test for related samples showed a statistically highly significant difference between the three measurement time points (χ^2^ = 17.3; df = 2; *p* < 0.001). The effect size according to Kendall (W = 0.719) indicated a large effect. Pairwise post hoc comparisons using Wilcoxon tests with Holm correction revealed statistically significant reductions from t0 to t1 (*p* = 0.008, corrected) and from t0 to t2 (*p* = 0.008, corrected), while no statistically significant difference was found between t1 and t2 (*p* = 0.656). The results demonstrate that both CCDs and DCDs led to significant improvements compared to baseline but did not differ significantly from each other.

The paired Wilcoxon test comparing dental evaluations between CCDs and DCDs showed no statistically significant difference (V = 34; *p* = 0.531). The effect size was small (r = 0.162). The median confidence interval of the difference included zero (−1.0 to 7.0), indicating no practically relevant difference between the prosthesis types.

## 4. Discussion

The present randomized controlled clinical pilot trial investigated patient satisfaction and clinical effectiveness of CCDs and DCDs in 15 edentulous patients. The objective was to evaluate whether CCDs or DCDs achieve superior OHRQoL values and clinical performance. The results demonstrate that both fabrication methods led to significant improvements in OHRQoL compared to the edentulous state. While objective clinical parameters showed no significant differences between groups, subjective evaluations indicated a slight advantage for DCDs.

Regarding clinical effectiveness, studies have demonstrated that CCDs and DCDs achieved comparable clinical results [14,16], sometimes with slight benefits toward DCDs but with reduced patient numbers [16]. The same results were obtained in the present clinical study. However, the small numerical difference of 3.0 points favoring DCDs in our study is noteworthy. This small numerical advantage may be explained by several factors inherent to digital workflows. Digitally fabricated complete dentures are milled from prepolymerized resin blanks, which may result in improved base adaptation, reduced polymerization shrinkage, and more consistent reproduction of the planned denture geometry. These factors may contribute to enhanced initial comfort, perceived stability, and retention, which are known to influence patient-reported outcomes such as OHRQoL. With regard to clinical relevance, it should be noted that no universally accepted minimal clinically important difference has been established for the OHIP-G49 in edentulous populations. Available literature suggests that the observed numerical difference of 3 points likely falls below thresholds generally considered clinically meaningful. Therefore, this difference should be interpreted as a trend rather than a clinically relevant superiority. Although all clinical evaluations were performed by a single experienced examiner to ensure intra-examiner consistency, no formal examiner calibration or inter-examiner validation was performed. Therefore, potential assessment bias cannot be fully excluded, and the external validity of the clinical scoring may be limited.

In addition, the digital workflow allows for a more standardized and reproducible fabrication process, potentially reducing minor inconsistencies that can affect patient perception, even when objective clinical parameters are comparable. However, given the small sample size and the absence of statistically significant differences, this numerical advantage should be interpreted with caution. Minor post-insertion adjustments were required in both CCD and DCD groups during the initial follow-up period. The frequency and extent of adjustments were comparable between fabrication methods and primarily involved pressure relief and occlusal refinement.

Clinician-reported outcomes varied depending on the digital technique [14]. Milled dentures demonstrated higher clinician satisfaction and required fewer post-insertion adjustments than CCDs, while 3D-printed dentures performed similarly to traditional ones [14]. While these results support the use of CAD/CAM technology as a clinically viable and cost-efficient alternative to conventional fabrication, the current body of evidence remains limited [14]. Kattadiyil et al. reported significantly higher ratings for DCDs regarding denture base contour, fit, extension, stability, retention, and overall outcome, whereas no significant differences were identified for tooth arrangement, esthetics, lip support, occlusion, phonetics, centric relation, vertical dimension of occlusion, or prognosis [35]. The greater retention observed in maxillary DCDs may be attributed to the elimination of polymerization shrinkage and the fabrication of prostheses from prepolymerized acrylic resin blocks using a milling process. However, these findings require validation through objective measurement [35]. Peroz et al. reported that the dimension of the denture border is dependent on the fabrication method [24]. Regarding DCDs, the denture border was significantly more frequently overextended. Consequently, retention was reduced immediately after insertion of the dentures in the upper jaw. However, this parameter could be corrected, and subsequently no clinical difference between CCDs and DCDs was demonstrated [24].

Regarding patient satisfaction, the significant improvement in OHIP-G49 scores observed in the present study, from a median baseline value of 70.0 to 19.0 (CCDs) and 16.0 (DCDs), is consistent with previous investigations. Peroz et al. found in their randomized controlled trial that the fabrication method of complete dentures had no significant impact on OHRQoL, confirming our observation that both fabrication methods result in comparable quality of life improvements [13].

However, the small numerical difference of 3.0 points favoring DCDs in our study is noteworthy. Peroz et al. also utilized the OHIP-G49 to compare DCDs and CCDs and found that patients with CCDs experienced fewer functional limitations and less physical pain than those with DCDs two weeks after insertion [13]. However, it should be noted that it was a double-blind study that was conducted. These contradictory findings underscore the heterogeneity of available evidence and the need for larger-scale studies.

Casucci et al. reported that DCDs achieved comparable clinical outcomes to CCDs, with no significant differences in bite force, masticatory performance, or patient satisfaction (OHIP-G14) [40]. Their key advantages were reduced chairside time (154.3 ± 13.2 min vs. 218.0 ± 20.8 min; *p* < 0.0001) and lower laboratory costs (378.8 ± 137.5 € versus 459.2 ± 63.7 €; *p* = 0.0059). Between digital techniques, 3D printing proved more cost-efficient than milling [40].

Previous studies investigating digitally fabricated complete dentures have reported heterogeneous results with regard to clinical performance and patient-reported outcomes. While some investigations suggest advantages of DCDs in terms of retention, comfort, or patient satisfaction, others report comparable outcomes between digital and conventional fabrication methods. Moreover, many available studies are limited by small sample sizes, short observation periods, non-randomized designs, or the use of different outcome measures, which hampers direct comparability.

The present randomized controlled cross-over clinical trial adds to the existing body of evidence by directly comparing conventionally fabricated and milled digital complete dentures within the same patients using a standardized and validated OHRQoL instrument (OHIP-G49) combined with structured clinical evaluation criteria. By minimizing interindividual variability and evaluating both subjective and objective outcomes after defined adaptation periods, this study provides additional clinical insight into patient-centered effects of digital denture fabrication that remain insufficiently addressed in the current literature.

Regarding limitations of the present study, the small sample size of 15 patients must be acknowledged, as it limits the statistical power and generalizability of results. This exploratory randomized controlled clinical trial with pilot character included a limited number of patients. Therefore, the results should be interpreted with caution, and the findings primarily serve to generate hypotheses for future larger-scale randomized studies. Future studies should include larger cohorts to detect subtle differences between fabrication methods. The three-month observation period for each denture is relatively short for evaluating long-term performance. Extended observation times would be desirable for future investigations. However, comparable studies have also conducted follow-up periods of three months [13]. It should be acknowledged that the extended recruitment period (2015–2025) coincided with ongoing technological development in digital dentistry (software updates, scanner performance, CAD/CAM hardware and material properties).

Although a standardized workflow was maintained, subtle influences of technological evolution on the homogeneity of digitally fabricated dentures cannot be entirely excluded and may have influenced outcomes as a potential confounding factor.

The present study did not include cost analysis, although this represents an important factor in deciding between fabrication methods. Nevertheless, it has been established that DCDs are more economical due to reduced chairside time and lower laboratory costs [14,40]. Between digital techniques, 3D printing is more cost-effective than milling. Overall, digital workflows represent a practical and efficient alternative, supporting wider application in healthcare systems. Another limitation of the present study is that, although patients were blinded to the denture fabrication method, clinicians performing the denture evaluations were aware of the prosthesis type. This may represent a potential source of observer bias, particularly with regard to clinician-based evaluation scores. This limitation should be considered when interpreting the objective clinical assessment results.

Despite standardized administration, patient-reported outcome measures such as the OHIP-G49 are inherently subject to potential sources of bias, including subjective interpretation of items and individual response behavior. The loss of questionnaire data for two participants reduced the sample size available for longitudinal OHRQoL analysis and may have introduced attrition bias, particularly given the small overall cohort. These limitations should be considered when interpreting the results.

A significant advantage of DCDs is the preservation of digital data, which can be invaluable for future repairs or replacement fabrication, particularly for elderly patients [41,42,43]. This aspect was not considered in our short-term observation but should be factored into treatment planning. Available scientific studies have varied in design and quality, and long-term outcomes have been insufficiently addressed. Therefore, further well-designed randomized clinical trials are necessary to establish the clinical relevance of specific digital manufacturing approaches, particularly regarding durability, functional outcomes, and patient-centered measures.

## 5. Conclusions

The present study demonstrated that both CCDs and DCDs result in clinically acceptable outcomes. While DCDs show slight advantages in subjective evaluation and clear advantages in treatment efficiency, both methods are suitable for rehabilitating edentulous patients. The choice of fabrication method should be made individually, considering patient preferences, clinical circumstances, and available resources. The findings of the present exploratory randomized controlled clinical pilot trial should be interpreted as a pilot study with limited statistical power.

Future research should focus on larger, multicenter, long-term studies that include extended follow-up periods alongside the collection of comprehensive clinical parameters to develop evidence-based recommendations for clinical practice.

## Figures and Tables

**Table 1:** Demographic data of the study sample.

Parameter	Value
number of patients (n)	15
age (years) mean ± standard deviation	69.8 ± 8.7
age (years) median (range)	69 (58–83)
sex male	7 (46.7%)
sex female	8 (53.3%)

**Table 2:** Classification of OHIP-G49 sum scores into evaluation categories (highly satisfactory, satisfactory with limitations, unsatisfactory). Higher OHIP-G49 scores indicate a greater negative impact on oral health-related quality of life.

Points	Evaluation Category
0–82	Highly satisfactory
83–164	Satisfactory with limitations
165–245	Unsatisfactory

**Table 3:** Clinical criteria used to evaluate the functional and esthetic performance of complete dentures [38,39]. Each criterion was rated on a 5-point scale, with higher scores indicating poorer outcomes.

Criterion	Description
Retention	Ability of the denture to remain in place through adhesion and cohesion; prevents dislodgement during function.
Static occlusion	Tooth contacts in maximum intercuspation without mandibular movement; contributes to even load distribution.
Muscle control	Capacity of surrounding musculature (tongue, lips, cheeks) to maintain denture stability during function.
Outline form of the base	Shape and border extension of the denture base; should accommodate soft tissue dynamics.
Tooth positioning	Spatial arrangement and angulation of artificial teeth; influences function and esthetics.
Fit (Adaptation)	Degree of adaptation of the denture base to mucosal tissues; associated with comfort and retention.
Esthetics	Natural appearance of teeth and support of facial structures; affects patient satisfaction.
Dynamic occlusion	Tooth contacts during mandibular excursions; relevant for functional stability.
Lip support	Contribution of denture and anterior teeth to lip contour; affects esthetics and phonetics.
Vertical dimension	Interarch distance at occlusion; important for function, appearance, and TMJ health.
Extension	Coverage of the denture base; should be maximized without impinging on movable tissues.
Stability	Resistance to horizontal and rotational forces; prevents displacement of the denture.
Phonetics	Influence of the denture on speech articulation and clarity.

## Data Availability

The original contributions presented in this study are included in the article and Supplementary Material. Further inquiries can be directed to the corresponding author.

## Supplementary Materials

The following supporting information can be downloaded at: https://www.mdpi.com/article/10.3390/dj14040221/s1, Table S1: CONSORT 2025 checklist [37].

## Abbreviations

The following abbreviations are used in this manuscript:

CONSORT	Consolidated Standards of Reporting Trials
CAD/CAM	Computer-aided design/computer-aided manufacturing
OHRQoL	Oral health-related quality of life
DCD	Digitally fabricated complete denture
CCD	Conventionally fabricated complete denture
PMMA	Polymethylmethacrylate
IQR	Interquartile range
